# Notes from Professor Kent’s Lecture upon Ignatia

**Published:** 1885-12

**Authors:** 


### NOTES FROM PROFESSOR KENT'S LECTURE UPON IGNATIA.

This is the old women's and the nervous girls' remedy—concomitant phenomena which are contradictory to or inconsistent with each other. There is a wonderful state of weakness, alternating with a wonderful condition of strength, which is found in hysterical women; states and conditions brought about by anger and grief; hysterical aphonia; paralysis of either hand, that comes and goes,—these opposite conditions guide you in a general way to Ignatia; great grief after loss of friends or persons that are very dear (Natrum mur. follows if Ignatia does not cure); children who have been reprimanded and sent to bed become sick; ailments from grief, mortification, and bad feeling; effects of disappointed love; throbbing pain in the occiput; sensation as if a nail were driven into the side of the head—better when lying upon it; the sore throat of Ignatia is better when swallowing solids; flickering zigzag before the eyes—this occurs in nervous states after grief, anger, or disappointed love; many of the eye symptoms are very deceiving; headache from coffee, tobacco, or stimulants—they even dread the smell of coffee; a lady will get sick-headache when in a room where there is tobacco smoked.

There are all sorts of spasmodic twitching of the facial muscles. It is a great remedy for spasms, stitches in the soft palate extending to the ear, stitches in the throat only between the acts of swallowing; the patient obtains relief from swallowing, (Zincum, has it in a less degree); sensation of a lump in the throat when swallowing; choking sensation as of a lump rising from the stomach (globus hystericus; the Lach. ball goes down). Ignatia in that is something like Nux vomica, but Nux is not useful for hysterical women. Ignatia is also worse on empty deglutition. Hunger and nausea at the same time; feeling of hunger in the evening, which prevents sleep; desire for various things, which are refused when offered (Bry.). Sometimes a hysterical person when vomiting will take a fit—the character of the vomiting is of little consequence—a handful of cold-slaw or some vinegar will relieve; sensation of weakness and sinking in pit of stomach. "All-gone" sensation, with sighing, calls for Ignatia only. Some of the very worst cases of hæmorrhoids may be treated with Ignatia—the pain running up into the rectum and bowels, the piles being either bleeding or blind. Ignatia produces a very troublesome constipation and diarrhœa, which may be either mucous or bloody or slimy; menses too soon, scanty, or profuse—the blood is as black as tar, of a putrid odor, and clotty; hemorrhage from Chamomile tea; cramping pain in uterus, worse from touching the parts. This spasmodic state may be somewhat hysterical. It is a great remedy during pregnant state for puerperal convulsions and vomiting; for after-pains with much sighing, and perhaps globus hystericus and "all-gone" feeling in stomach. American and French women will usually require Ignatia for nervous conditions, while Nux vomica is the most suitable for English women. Ignatia is one of the most useful remedies in laryngismus stridulus (Gels. and Laur.); desire to take a deep breath; sighing; oppression of the chest—so great she thinks she has the heart-disease; coughs every time he comes to a stand-still during a walk. A nervous girl begins to cough in bed at night; coughs until she sweats, and the more she coughs the more she sweats, and finally she becomes exhausted from coughing. The longer she coughs the more the irritation of the cough increases. After a spasm the Ign. patient will become perfectly rigid; paralysis after great mental emotion and night-watching in a sick-chamber; chorea or chronic spasms, after fright, with grief; worse after eating; better when lying on the back. The Ign. patient will be perfectly wide awake one instant and fall asleep the next (Baptisia has it from congestion of the brain). The Ign. patient is generally jealous (like Apis and Hyos.). The chill, fever, and sweat is important and just as deceptive; chill and thirst better from external warmth (Apis and Arnica). As soon as patient gets near the stove the chill passes away, and *vice versa*. There is no thirst during the heat; internal chill, external heat, and *vice versa;* external heat, without thirst, with intolerance of warmth, but relief during the chill from warmth. The apyrexia will be attended by an "all-gone" feeling in the stomach; sensation as if sweat would break out—which does not follow. The patient is very sensitive to pain, contact, and injuries; generally worse from slightest touch, but better from hard pressure; symptoms worse from drinking water, because of the slight contact. Ignatia is similar in some respects to Zincum; they antidote each other (also Ign. and Coffee). As a general thing, the Ignatia patient is fine-grained, high-toned, and easily disturbed; sometimes tearful, but not in the same sense that Puls. and Apis are. The Ignatia patient is absent-minded, and will alternately cry and laugh.

